
JOURNAL INFORMATION
==============================
NLM Title Abbreviation: ScientificWorldJournal
Journal ID: ScientificWorldJournal
NLM Journal ID: 101131163
Journal ID: TSWJ

The Scientific World Journal

ISSN: 2356-6140
EISSN: 1537-744X
Publisher: Wiley

ARTICLE INFORMATION
==============================
PMCID: PMC6009446
PMID: 29973812
DOI: 10.1100/tsw.2002.38
Article ID: 403457
Article version: 1
Subjects: Short Report

Prediction of C Regulatory Elements Using Statistical Analysis

Qiu Ping *
Ding Wei
Jiang Ying
Greene Jonathan
Wang Luquan
Bioinformatics Group and Human Genomic Research Department at Schering-Plough Research Institute, 2015 Galloping Hill Road, Kenilworth, NJ 07033, UK
*Ping Qiu: ping.qiu@spcorp.com
Publication date: 2002
Electronic publication date: 2002 May 8
Volume: 2
First page: 79
Last page: 81
Copyright: Copyright © 2002 Ping Qiu et al.
Copyright year: 2002
License: This is an open access article distributed under the Creative Commons Attribution License, which permits unrestricted use, distribution, and reproduction in any medium, provided the original work is properly cited.
License URL: https://creativecommons.org/licenses/by/3.0/

==============================
Advances in Gene Technology: The Genome and Beyond –
Structural Biology for Medicine (Proceedings of the 2002 Miami
*Nature Biotechnology* Winter Symposium)
*TheScientificWorld* 2002, 2(S2), 79–81
ISSN 1532-2246; DOI 10.1100/tsw.2002.38

### PREDICTION OF C REGULATORY ELEMENTS USING STATISTICAL ANALYSIS

Ping Qiu*, Wei Ding, Ying Jiang, Jonathan Greene, and Luquan Wang
Bioinformatics Group and Human Genomic Research Department at Schering-Plough Research Institute, 2015 Galloping Hill Road, Kenilworth, NJ 07033
* ping.qiu@spcorp.com

**INTRODUCTION.** Complex differential expression of genes in higher organisms is achieved through combinatorial regulation of transcription by specific combination of transcription factors binding to their target sites in the regulatory regions of these genes. The composite elements (CE) model was defined as pairs of closely situated binding sites, corresponding transcription factors, protein–protein interaction between them, and expression patterns provided by this combinatorial regulation[1]. In this study, we describe a novel computational approach to detect possible composite elements in genomic sequence. The method is based on the detection of over-represented adjacent transcription binding sites.

**METHOD.** A computational transcript mapping approach was used to locate promoter sequences for GenBank RefSeq records within their genomic organization. The promoter reference database was validated by comparing with GenBank annotated promoters and has been described in a previous published paper[2]. These promoter regions were scanned for potential binding sites using the Matinspector program and the TRANSFAC[3] transcription binding site scoring matrix library. We used 20- and 50-bp as distance cutoffs to predict composite elements. The random frequency of two TF binding sites existing within 20- or 50-bp over a 2000-bp promoter sequence was calculated. The discrepancy between the actual frequency of the composite elements and the random frequency was evaluated by determining the statistical variable chi-square ($\chi 2$). The p value was further derived from chi-square.

**RESULTS.** For each gene, the genomic sequence 2000-bp upstream of the 5’ end of the mRNA was retrieved as promoter region. 163 human TF binding site matrices from TRANSFAC 4.4.2 were used for this analysis. Out of the 13203 possible combinations of any two TF matrices, 339 pairs coexist within 20-bp (DIS = 20) distance and are over-represented in the reference promoter database and has a $\chi 2$ value of 8 or above (matrix similarity score, MSS = 0.9). They account for 2.6% of the total possible combination.

We validated 46 CEs from COMPEL[1] which have binding sites matrix in TRANSFAC 4.4.2. Out of the 46 CEs, 18 of them were predicted with our method with $\chi 2$ values >= 8. Most of them show significantly high $\chi 2$ values (Table 1). It is interesting to know if our prediction can pinpoint to some real CEs which have not yet been collected by COMPEL. One direct approach would be to take some predicted CEs with extremely high $\chi 2$ value that are not in COMPEL and look for supporting information from the scientific literatures. By this approach, we have been able to confirm quite some, for example Pbx and Oct-1, C/EBP and SRF, YY1 and NF-AT, EGR and SP1 etc.

**TABLE 1**.
List of composite elements from COMPEL (Release 2.4) that can be predicted from *in silico* analysis.

| FACTOR1 | FACTOR2 | $\chi$2(MSS=0.9, DIS=20) | $\chi$2(MSS=0.9, DIS=50) | $\chi$2(MSS=0.8, DIS=20) | $\chi$2(MSS=0.8, DIS=50) |
|---|---|---|---|---|---|
| NF-Y | NF-1 | 490.47 | 330.69 | 93.39 | 310.50 |
| Sp1 | NF-Y | 8.05 | 67.55 | 10.97 | 48.97 |
| YY1 | SRF | 123.01 | 60.01 | 84.91 | 53.14 |
| COUP-TF | ER | 13.14 | 15.44 | 49.54 | 15.48 |
| HNF-1 | 1-Oct | 21.16 | 14.77 | 117.20 | 189.47 |
| CREB | HNF-1 | 87.39 | 35.16 | obs<exp | 0.03 |
| NF-kappaB | Sp1 | 8.51 | 5.64 | 6.50 | 27.90 |
| CREB/ATF family | NF-Y | 9.12 | 21.94 | 12.78 | 21.33 |
| C/EBPalpha | NF-Y | 3.89 | 14.60 | 380.43 | 275.67 |
| C/EBPbeta | HNF-1 | 3.83 | 8.00 | 22.68 | 72.90 |
| HNF-4 | COUP | 1698.5 | 676.79 | 363.70 | 101.44 |
| Sp1 | E2F-1 | 8.82 | 10.55 | 19.78 | 61.28 |
| C/EBPalpha | AML1 | 1263.94 | 578.93 | 1235.01 | 558.92 |
| C/EBPalpha | HNF-4 | 1.16 | 12.32 | 3.01 | 17.38 |
| RFX | CEBP/ATF family | 2.41 | 8.48 | obs<exp | 1.46 |
| HLH family | Octamer family | 0.01 | 24.95 | 0.15 | 29.94 |
| SP1 | c-ETS-1 | 0.01 | 0.01 | 1.12 | 17.82 |
| AP1 | c-ETS-2 | 0.89 | 0.16 | 13.26 | 0.07 |

**DISCUSSION.** By combining profiles of some relatively well-characterized regulatory elements with statistical significance analysis of their close-by coexistence, we have generated an efficient computational means of identifying CEs. It should be noted that some composite elements in COMPEL database were not identified using the parameters of the analysis presented here. Multiple reasons could account for this outcome. For example: (1) some TRANSFAC TF binding-site matrices are outdated in terms of quality and specificity, (2) a lower cutoff score for the matrix similarity score would increase the sensitivity but lower the specificity for some TFs, (3) the distance cutoff between the composite elements we used, 20- or 50-bp, might not reflect the actual distance for some composite elements etc. The performance of this prediction method described is sufficiently specific to warrant further analysis of predicted composite elements. This prediction algorithm might also help to supplement COMPEL or other similar database since it can efficiently point to high quality putative composite elements.

### REFERENCES

1. Kel, O.V., Romashchenko, A.G., Kolchanov, N.A., Wingender, E., and Kel, A. (2000) Nucl. Acids Res. 28, 311–315.
2. Wingender, E., Chen, X., Fricke, E., Geffers, R., Hehl, R., Liebich, I., Krull, M., Matys, V., Michael, H., Ohnhauser, R., Pruss, M., Schacherer, F., Thiele, S., and Urbach, S. (2001) Nucl. Acids Res. 29, 281–283.
3. Wang, L., Wu, Q., Qiu, P., Mirza, A., McGuirk, M., Kirschmeier, P., Greene, J.R., Wang, Y., Pickett, C.B., and Liu, S. (2001) J. Biol. Chem. 276, 43604–43610.
